# Land Use Mix in Functional Urban Areas of Selected Central European Countries from 2006 to 2012

**DOI:** 10.3390/ijerph192215233

**Published:** 2022-11-18

**Authors:** Dawid Kudas, Agnieszka Wnęk, Lucia Tátošová

**Affiliations:** 1 Faculty of Environmental Engineering and Land Surveying, Department of Geodesy, University of Agriculture in Krakow, al. Mickiewicza 21, 31-120 Kraków, Poland; 2 Faculty of Horticulture and Landscape Engineering, Institute of Landscape Engineering, Slovak University of Agriculture in Nitra, Tr. A. Hlinku 2, 949 76 Nitra, Slovakia

**Keywords:** land use, land use mix, functional urban area, entropy, dissimilarity index

## Abstract

The land use mix (LUM) is among the critical issues in spatial planning because it can determine the occurrence and structure of various land use and cover types (LUC) and prevent any adverse patterns. The paper focuses on the LUM in functional urban areas (FUAs) in Czechia, Slovakia, Poland, and Hungary. The research employed Urban Atlas (UA) data on LUC in 2006 and 2012 to characterise LUM in the FUAs. The research follows the division of the FUA into the urban area (urban core, UC) and its functional surroundings (commuting zones, CZ). We further characterised the phenomena investigated for the entire country, region, and Europe using Corine Land Cover (CLC) data. The LUM was quantified with the entropy index (EI), dissimilarity index (DI), and multi-dimensional balance index (MBI). The EI demonstrated that the investigated FUAs went through more substantial LUM changes than the 27 European Union member states (EU27) from 2006 to 2012. Moreover, it has been demonstrated that LUM overspill was more intensive in CZs than in UCs on the regional and national levels. We found out that urbanised areas grew at the expense of agricultural areas in both UCs and CZs with similar dynamics in 2006–2012 in all the analysed countries.

## 1. Introduction

According to the data of the statistical office of the European Union (Eurostat), the European population has grown substantially in recent years. It is forecast to stabilise or even decline in the coming years. Research by Ref. [1] for the Organisation for Economic Co-operation and Development (OECD) demonstrated that populations in areas around cities tend to grow, while more remote regions lose residents. Therefore, an increase in population will directly affect urbanised and agricultural land cover in suburban zones. For instance, as the population in Europe grew mostly in cities, the area of agricultural land declined in favour of woodlands [2]. As reported by Antrop [3], urbanisation defined as the percentage of the population living in cities has recently increased sharply up to 80% in most European countries. The economic transformation of recent decades consisted of a shift from agricultural to industrial production and an increase in productivity, which led to a universal loss of agricultural land. Furthermore, an increase in population and continuous urbanisation growth contribute to changing land use patterns and urbanscape [4].

The dynamics of land-use changes are more intensive in post-socialist countries. This phenomenon occurs globally [5,6] and in Europe, particularly Central Europe [7,8]. Experts attribute the European dynamics to the breakdown of socialism in 1989 and ensuing changes in ownership law, decollectivization, and abandonment of agriculture [9,10,11]. Abandoned intensive agricultural land was usually transformed into grassland and forests. Furthermore, Central Europe, while believed to be less urbanised, has undergone a dynamic, if somewhat uncontrolled, growth of urban areas in suburban zones in recent decades [12]. As a result, agricultural land fragmentation is most extensive in suburban zones.

Central European countries where socialism broke down include Czechia, Hungary, Poland, and Slovakia. The period of 1990–2010 saw a rapid increase in built-up areas in Czechia, especially in large agglomerations [13]. Stych et al. [14] identified a gradual growth in the spatial diversification of built-up areas in Czechia over the last century and a concentration of half of the built-up areas on 21% of the country’s territory in 2010. Urbanisation was not the dominant land-use change in Slovakia. Nevertheless, in relative terms, it was among the key land-related processes, mostly in the lowlands [15]. At the same time, urbanisation in Slovakia declined from the fall of socialism to its accession to the European Union (EU) in 2004 followed by a 0.25% annual increase in urbanisation until 2012 [15]. Furthermore, structural changes in the agrarian landscape occurred, where the share of arable land and grassland changed [16]. In Poland, urbanised areas have been growing regularly since 2000, with built-up areas expanding mostly in urban areas [17]. Urbanised areas flourish in functional urban areas (FUAs), commuting zones (CZs) and slightly less in urban cores (UCs) [17]. OECD believes these changes are caused mostly by population movement from UCs to CZs. Wnęk et al. [8] also identified an increase in urbanised land at the expense of agricultural land from 2006 to 2012 in Polish FUAs. Land use per capita has grown in Hungary since 2000 in urban, rural, and suburban areas due to shrinking population and continuous land development [18].

**Figure 1:**
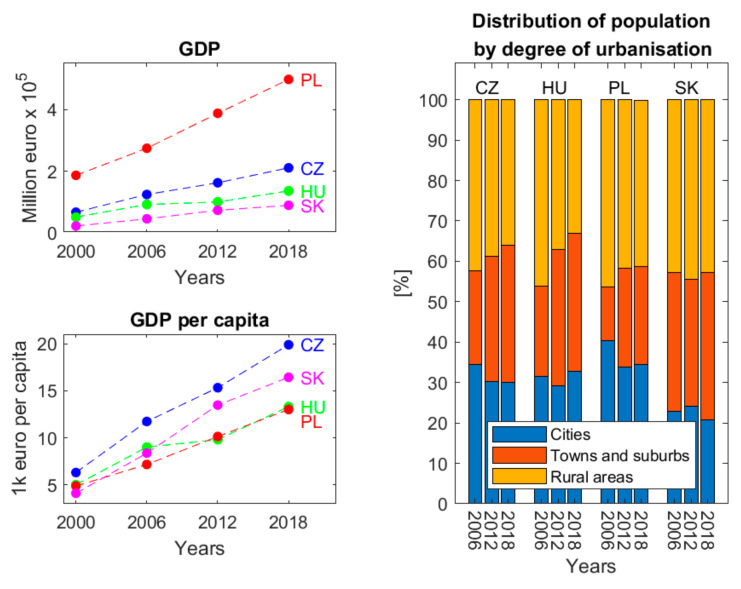
Summary of the GDP, GDP per capita, and the distribution of the population by the degree of urbanisation in 2000–2018 in Czechia, Hungary, Poland, and Slovakia. Please note data on the distribution of the population by the degree of urbanisation are missing for the year 2000 (Source: own elaboration based on Eurostat data [19]).

The countries investigated differ in terms of their populations. Czechia and Poland have the greatest population densities with about 132 and 118 people/km^2^, respectively. Slovakia and Hungary have approx. 110 people/km^2^. In Czechia, Hungary, and Poland, the total share of people living in cities, towns, and suburbs grew to nearly 70% from a little over 50% between 2006 and 2012 (Figure 1). Slovakia is an exception in this regard with a 0.6% increase in rural population from 2006 to 2012 followed by a decrease by 0.7% to 42.7% in 2018. In terms of gross domestic product (GDP), Poland achieved an evident increase, and Czechia and Slovakia a slightly lesser growth from 2000 to 2018 (Figure 1). At the same time, all these states improved their GDP per capita from 2000 to 2018, with Czechia and Slovakia leading the pack (Figure 1).

The adverse impact of urban sprawl can be controlled with the proper land use mix (LUM). A complementary mix of various types of land use categories can be beneficial to human life quality and health [20]. The LUM has recently become an important tool in various tiers of spatial planning. LUM indicators are a way to quantify combinations of land-use patterns in an area. The eight most popular of them are the entropy index (EI), dissimilarity index (DI), Herfindahl-Hirschman index, balance index (BI), clustering index, Gini index, exposure index, and Atkinson index. According to Jao et al. [21], the EI is a popular measure in LUM research. It is an integral measure sensitive only to the aggregate distribution of land use types in the analysed area [22]. However, the EI often remains insensitive to many critical differences in spatial patterns and is unsuitable as an urban sprawl measure [23]. Therefore, an EI characteristic of an area is supplemented with the DI, considered a divisional measure. The DI can determine how much the distribution of various land use types in a part of an area is similar to the distribution in the entire area [22]. Both EI and DI can quantify land use mixing [24,25]. Another measure employed in LUM studies is the BI, which is most often calculated for two distinct categories to investigate their balance in an area, demonstrating the dominance of one of them or an equilibrium. In the case of LUM research involving more LUC classes or categories, one can employ a generalised BI, multidimensional balance index (MBI) [22].

We investigated the LUM in 62 FUAs in Czechia, Slovakia, Poland, and Hungary in 2006 and 2012 using the EI, MBI, and DI diversity indices. The research and ensuing discussion provide valuable insight into the local LUM in individual FUAs and also in their UCs and CZs. Quantitative research will identify general trends and potential extremes that require more sophisticated LUM spatial analyses. We further touched upon LUM changes on a national level with analyses of changes in the aggregate FUA area in a country and on a regional level for a total area of all FUAs. Apart from providing valuable input for spatial planning and spatial strategies, the research will answer the question of whether the LUM in individual national FUAs is different than in the other countries. This will verify the hypothesis that countries with similar socioeconomic and historical backgrounds exhibit similar trends in land use change dynamics, particularly in FUAs.

## 2. Materials and Methods

### 2.1. Study Area and Data

There are many sources of data on land use and cover (LUC) in Europe, and one of which, Copernicus, is the European Union’s Earth Observation Programme. It consists of the CORINE Land Cover (CLC) database and the Urban Atlas (UA). CLC regularly provides information on 44 LUC classes for 1990, 2000, and then in six years. The CLC minimum mapping unit (MMU) for areal phenomena is 25 ha. In case of CLC data, geometric accuracy is better than 100 m, and thematic accuracy is ≥85%. The UA also has been providing regular updates on LUC since 2006 but only for FUAs. In the UA, LUC data are available for FUAs over a specific population threshold. In 2006, the data were available for FUAs with over 100 thousand people, while in 2012, the threshold was 50 thousand. The UA divides LUC into 17 urban classes with an MMU of 0.25 ha and 10 rural classes with an MMU of 1 ha. In the case of UA data, the positional pixel accuracy is ±5 m and minimum overall accuracy for class 1 (Artificial surfaces) is ≥85%, whereas minimum overall accuracy for all classes is ≥80%. The UA offers a better spatial resolution of data compared to CLC. UA has also 2018 LUC data, but an initial verification demonstrated that the expected accuracy was not reached and must be improved.

We used UA data on LUC from 2006 and 2012 (referred to as UA2006 and UA2012) to characterise the LUM in FUAs in Czechia, Hungary, Poland, and Slovakia [26,27]. Regarding the entire countries, regions, and Europe, we employed CLC data for 2006 and 2012 (referred to as CLC2006 and CLC2012) [28,29].

**Figure 2:**
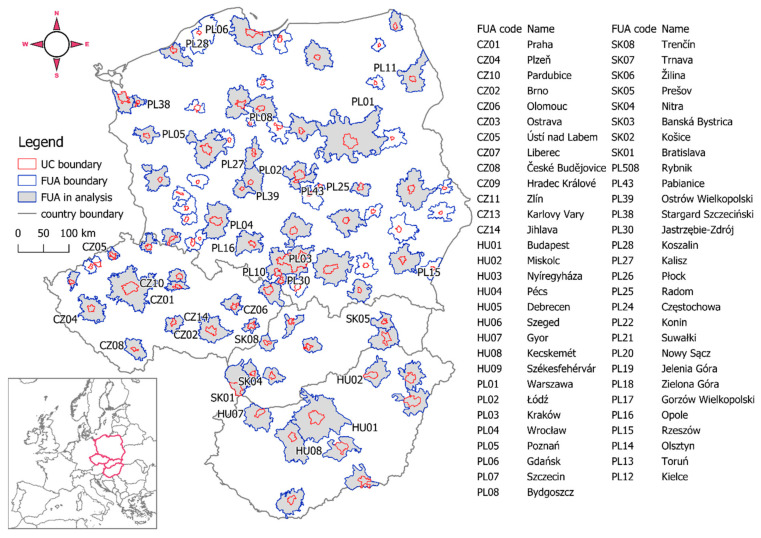
Location and designations of the FUAs in Poland, Czechia, Hungary, and Slovakia.

To compensate for differences in some FUA boundaries between UA2006 and UA2012 data, we used the intersect of each FUA (overlapping part). Areas labelled as class 91000 and 92000 (no LUC data) were excluded from the FUA intersects. As the FUA is defined as consisting of the urban core (UC) and its surroundings (CZ) containing less populated local units constituting the city’s labour market [30], the research considered the division. UCs and CZs were delimited using UC boundaries in UA2012 [27]. Sixty-two FUAs were investigated: 32 in Poland, 13 in Czechia, 9 in Hungary, and 8 in Slovakia (Figure 2). The total study area (FUAREG) constituted approx. 3% of the area of EU27.

The total FUA area for Poland was 6,035,823 ha (FUA_PL_), for Czechia 1,627,346 ha (FUA_CZ_), for Hungary 2,197,940 ha (FUA_HU_), and for Slovakia 867,346 ha (FUA_SK_). The area of FUAs representing each country amounts on average to approx. 20% of the area of the respective country. With data available for 2006 and 2012, the study area can be divided into two subzones, UC and CZ. UCs constitute 9.55% of FUA_PL_, 11.62% of FUA_CZ_, 11.87% of FUA_HU_, and 12.90% of FUA_SK_. The aggregate FUA and UC/CZ areas in each country were used to calculate LUM statistics on the national level. In the case of the regional level, we employed FUA_REG_.

The analyses involved major LUC classes as in UA2006: 1—artificial surfaces, 2—agricultural areas, semi-natural areas, and wetlands, 3—forests, and 5—water. To compensate for different LUC classifications in UA2006 and UA2012 but also CLC2006 and CLC2012, we aggregated detailed classes into major classes (Table 1). LUC classes are listed, described, and subdivided in the UA Mapping Guide [31].

### 2.2. LUM Measures Employed in the Study

LUM studies commonly use integral and divisional diversity indices, which facilitate holistic characterisation of the LUM when taken jointly. Available literature [22] demonstrated a strong correlation between values of some diversity indices used to characterise LUM. Hence, it is sufficient to use selected measures. The present study employed the EI and DI to assess the LUM and MBI to assess the LUM against a reference model.

### 2.3. Entropy Index (EI)

The EI is a diversity index ranging from 0 to 1 [32]. Low EI values reflect homogeneous land use, while higher values mean heterogeneous land use. The present research employs the EI to determine the LUM from the shares of several types of land use in an area given with an equation [22]:

(1) $$\mathrm{EI}=-\frac{\sum_{j=1}^{k}P_{j}\ln P_{j}}{\ln k}$$

where *k* is the land-use class in an area, *P_j_* is the percentage of land use *k* in the area. The EI is symmetrical in relation to land-use classes.

### 2.4. Dissimilarity Index (DI)

The DI can determine the degree of aggregation or segregation of various land-use types. Land-use diversification in a unit area can be determined with the generalised index of dissimilarity proposed by Sakoda [33]. We employed the dissimilarity index equations as proposed by Tivadar [34]:

(2) $$\mathrm{DI}=\frac{1}{2TIs}\sum_{k=1}^{4}\sum_{i=1}^{n}\left(t_{i}\left|p_{i}^{k}-P^{k}\right|\right)$$

(3) $$Is=\sum_{i=1}^{n}\left[\frac{t_{i}\left|p_{i}^{k}-P^{k}\right|}{2TP^{k}\left(1-P^{k}\right)}\right]$$

where *t_i_* is the total area of all land-use classes in spatial unit *i*; $p_{i}^{k}$ is the proportion of land-use class *k* in spatial unit *i*; $P^{k}$ is the proportion of land-use class *k* in the total area; *T* is the total area of all land-use classes in the total area; *Is* is the segregation index; *n* is the number of units delineated in the total area.

The DI assumes values from 0 to 1, where 0 is no dissimilarity and 1 means complete dissimilarity. As reported by Sun et al. [35], DI < 0.3 is conventionally interpreted as low diversity, 0.3 < DI < 0.60 means moderate diversity, and DI > 0.6 is high diversity. The dissimilarity index was computed in R Project using the OasisR library [34].

### 2.5. Multidimensional Balance Index (MBI)

The BI is calculated for two types of land use. When the number of land-use classes is greater, a generalised BI, MBI should be used instead [22]. MBI computations employ a model of land use class structure which is often determined by analysing land-use percentage in a unit larger than the investigated area. The model defines the reference value for the computed MBI value and should be balanced well. The MBI is calculated with the following formula:

(4) $$\mathrm{MBI}=1-\sum_{k}t_{k}\left|r_{k}-t_{k}\right|$$

where *t_k_* is the percentage of land-use class *k* in the reference area and *r_k_* is the percentage of land-use class *k* in a delineated subzone [22]. The MBI ranges from 0 to 1. High MBI values mean a correctly balanced LUM in the investigated area compared to the model LUM.

In the present paper, the *k* land-use type is the four major classes as defined and identified in the UA [31].

## 3. Results

### 3.1. LUC Percentages in the Investigated Areas

**Figure 3:**
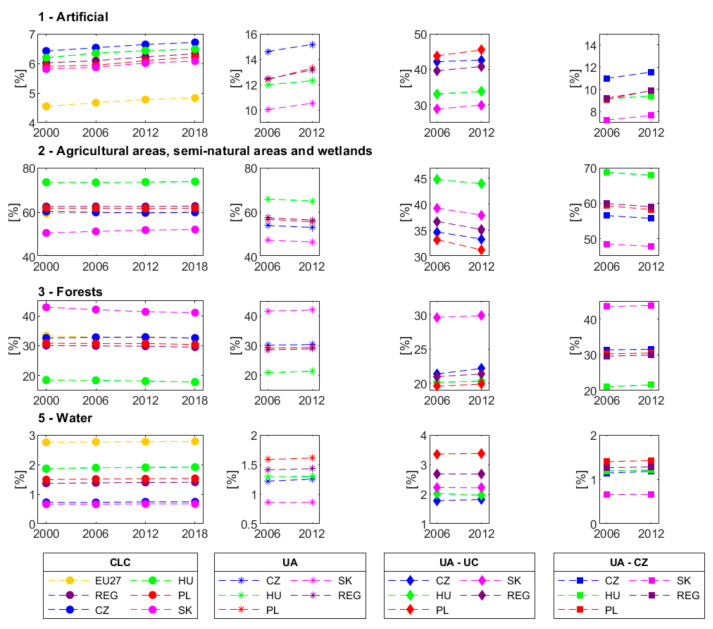
Changes in class percentages in the investigated countries, region (REG), and EU27 from 2000 to 2018 according to CLC data and for FUAs, UCs, and CZs for the period of 2006 to 2012 according to UA data. Please note differences in the scales of the vertical axes.

The largest area percentage in the FUAs area in 2006 and 2012 was occupied by class 2—agricultural areas, semi-natural areas, and wetlands: from 47.37 to 65.87% and from 46.57 to 65.04% of the LUM in 2006 and 2012, respectively. The second-largest type was class 3—forests, which occupied from 20.87 to 41.75% in 2006 and then from 21.36 to 42.04%. We noticed certain differences and similarities in the LUMs (Figure 3). In Slovakia and Hungary, the shares of class 2 in FUAs differed by about 18.5% and for class 3, by approx. 20.7%. Yet, the percentages of classes 2 and 3 were similar for Poland and Czechia. The most significant difference in class 1 was identified for Czechia and Slovakia. In each country, the percentage of classes 1, 3, and 5 increased, and class 2 declined in FUAs. Even though the increases and decreases in the LUM classes are consistent across the countries, Poland had an extraordinary growth of class 1.

In most countries, FUAs repeat the LUM identified with CLC for the entire country area with the most significant shift from class 2 to class 1. Another evident change is a positive trend of class 1 in the total country area and EU27 according to CLC data, which is still not as strong as for the FUAs. The LUM for the region of the investigated countries (REG) is similar to the LUM in Poland according to CLC data. The years 2006–2012 saw conversion of class 2 into classes 1 and 3 in FUA_REG_ by 0.70% and 0.29%, respectively. On the national level, class 1 grew, and class 2 declined in UCs and CZs. FUA_PL_ exhibited the largest increase in class 1 in UCs and CZs of 1.62% and 0.84%, respectively. The largest share of class 1 in CZs of over 11% was found in FUA_CZ_. All the investigated countries exhibited a slight increase in forests in UCs but no such change in CZs.

### 3.2. Local Changes

Changes in the EI, DI, and MBI were first analysed on a local level to investigate LUM changes in FUAs and their UCs and CZs.

**Figure 4:**
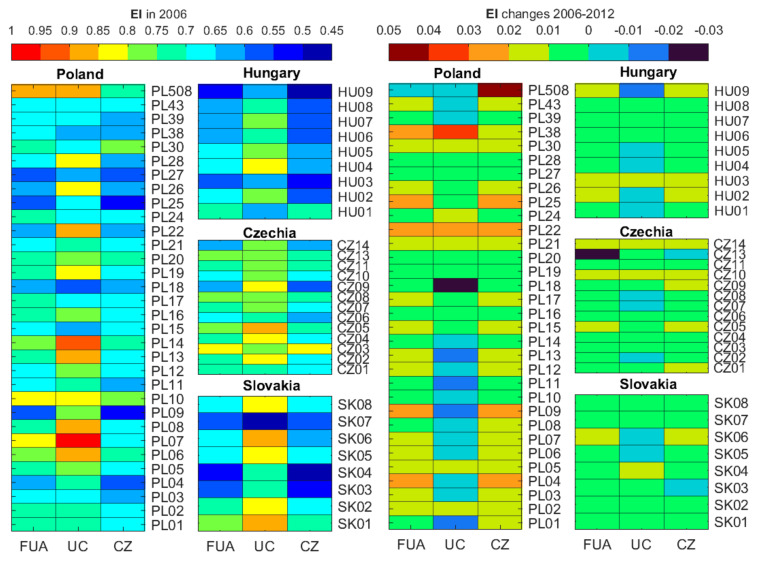
The entropy index in 2006 and its changes between 2006 and 2012 in the FUAs and their UCs and CZs in Poland, Slovakia, Czechia, and Hungary. Please note the scale for the EI and its changes has been adapted to its value range.

**Figure A1:**
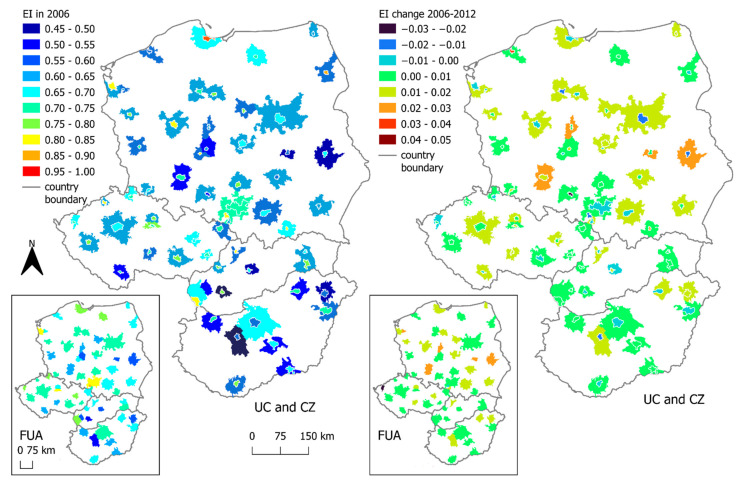
Maps of the entropy index in 2006 and its changes between 2006 and 2012 in the FUAs and their UCs and CZs in Poland, Slovakia, Czechia, and Hungary. Please note: the scale for the EI and its changes has been adapted to its value range.

In 2006, the EI in the FUAs ranged from 0.502 to 0.885. It was clearly greater for FUAs in Poland (0.558 to 0.885) and Czechia (0.627 to 0.821) than for the other countries. The EI for UCs demonstrated that the percentage share of the analysed LUC classes is closest to the uniform in PL06, PL07, PL08, PL13, PL14, PL22, PL508, SK01, SK06, and CZ05 (EI ≥ 0.850). The elevated EI values were recorded mainly for CZs in Poland and Czechia, just as was the case for FUAs. The EI in the investigated areas is shown in Figure 4 and Figure A1. From 2006 to 2012, the EI mostly increased in the FUAs by 0.001 to 0.024, with the exception of CZ13 and PL508. The index grew more in CZs than in UCs, which is evident in Poland. The EI dropped in 21 UCs out of the 62 FUAs, including in 13 UCs in Poland and 5 UCs in Hungary. The only CZs with a decrease in these years was CZ13. The EI increase in CZs ranged from 0.001 to 0.044. The EI grew on average by 0.009 in FUAs, 0.011 in CZs, and 0.002 in UCs with a similar standard deviation of around 0.007.

**Figure 5:**
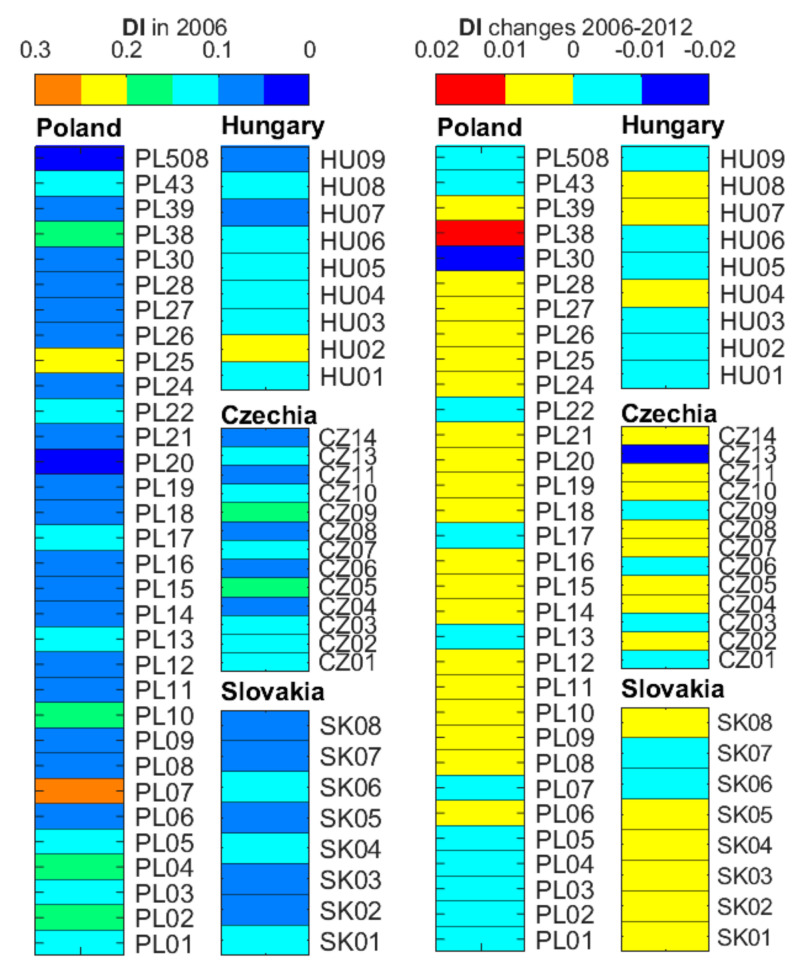
The dissimilarity index in 2006 in the FUAs, UCs, and CZs in Poland, Slovakia, Czechia, and Hungary. Please note: the scale for the DI and its changes has been adapted to its value range.

**Figure A2:**
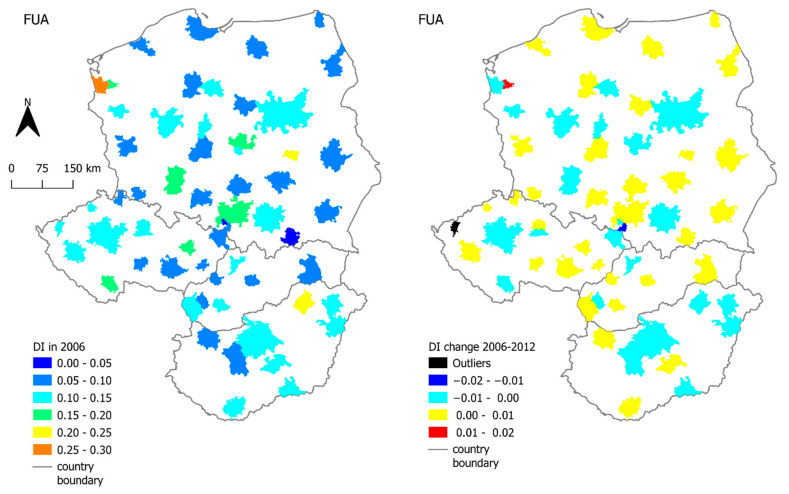
Maps of the dissimilarity index in 2006 in the FUAs, UCs, and CZs in Poland, Slovakia, Czechia, and Hungary. Please note: the scale for the DI and its changes has been adapted to its value range.

Figure 5 and Figure A2 show the DI for the FUAs. The largest DI values were identified for FUAs PL02, PL07, PL25, and HU02 (0.198, 0.286, 0.224, and 0.235 respectively). The DI did not exceed 0.2 in any of the FUAs, which is indicative of local LUM similarities in UCs and CZs. Changes in DI between 2006 and 2012 (Figure 5 and Figure A2) approximated 0, with the maximum value of 0.011 for PL38, 0.008 for SK04, and 0.007 for CZ14. The dissimilarity grew slightly there. The most evident reduction in dissimilarity was identified for PL30 and CZ13, –0.014 and –0.064 respectively. The low range of DI changes may be indicative of further blurring of differences in the LUM between UCs and CZs in the investigated period, with CZs increasingly resembling UCs.

**Figure 6:**
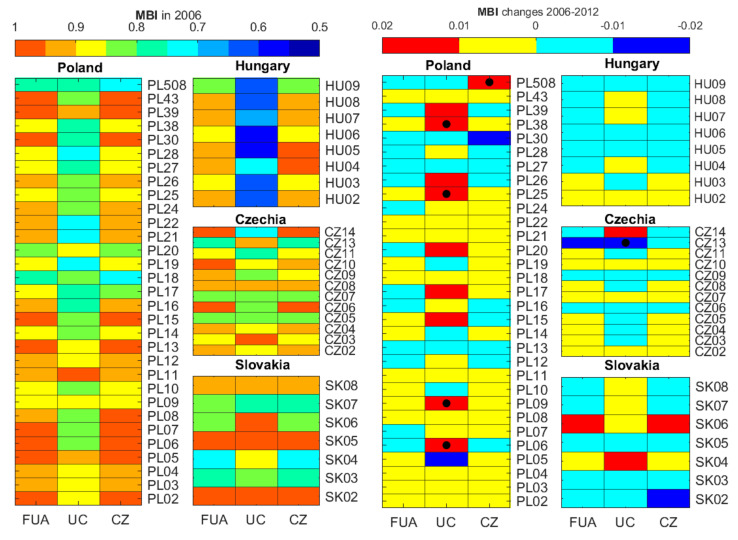
The multidimensional balance index in 2006 and its changes between 2006 and 2012 in the FUAs and their UCs and CZs in Poland, Slovakia, Czechia, and Hungary. Please note the scale for the MBI and its changes has been adapted to its value range and black dots indicate extreme values which are out of adopted range.

**Figure A3:**
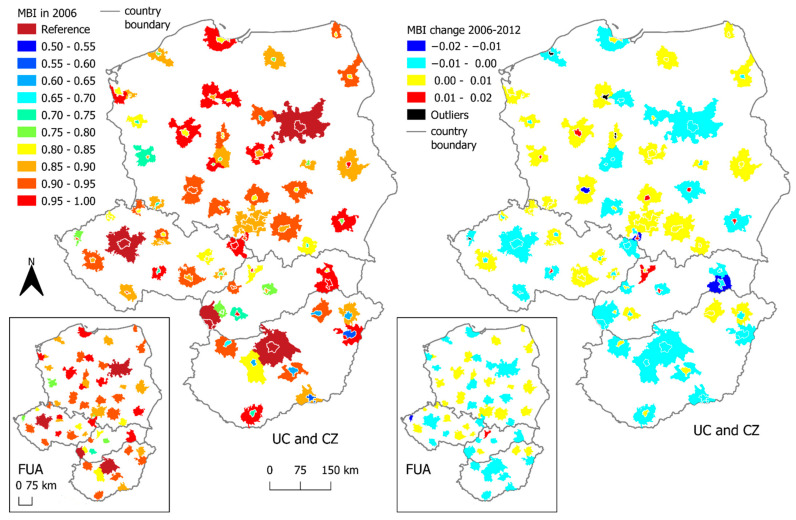
Maps of the multidimensional balance index in 2006 and its changes between 2006 and 2012 in the FUAs and their UCs and CZs in Poland, Slovakia, Czechia, and Hungary. Please note: the scale for the MBI and its changes has been adapted to its value range.

The value of the MBI in relation to the model defining the share of major classes in the capital FUAs and their UCS and CZs in 2006 is shown in Figure 6 and Figure A3. The model FUA is the capital of each respective country because it has the largest population. Hence, its LUM has been shaped under significant population pressure over the years. The FUA MBI in Hungary ranged from 0.831 to 0.946 in 2006. The index was much more varied in the other countries, from 0.741 to 0.993. Poland had 10 FUAs, Slovakia 2 FUAs, and Czechia 3 FUAs the 2006 MBI of which indicated a similar balance as in the relevant LUM model (BI ≥ 0.950). CZs appear to be better balanced than UCs in relation to the model, especially in Hungary. The MBI declined in 25 FUAs, 6 UCs, and 21 CZs from 2006 to 2012. The MBI changes are not homogeneous throughout the analysed areas (–0.012 to 0.013 for FUAs, –0.123 to 0.034 for UCs, and –0.013 to 0.027 for CZs). Extreme MBI changes from 2006 to 2012 were identified in the UC of FUA CZ13 (–0.123) and the CZ in FUA PL508 (0.027). CZ13 is a famous Czech SPA, in which UC forests grew significantly during the analysed period. We identified an improved LUM balance in relation to the model in most UCs in Poland and a decline in Czech UCs from 2006 to 2012. An improved balance reflected in the increased MBI may be indicative of a LUM shift towards a LUM typical of capital cities caused by a growing population.

### 3.3. National and Regional Changes

We then proceeded to analyse the LUM on the national and regional level to offer a generalisation of land-use changes. The values of the EI, DI, and MBI on the national and regional levels are provided in Table 2.

The EI grew on a national level from 2006 to 2012. The value of the index differed for each country, ranging from 0.658 to 0.742 in 2006 and from 0.666 to 0.749 in 2012. The EI assumed larger values for UCs than for CZs in both years, but the increase is apparently greater in CZs (Table 2). The largest increase in the EI was identified in Polish FUAs and their CZs, which is indicative of LUM changes there. We found the EI for FUA_REG_ to be generally constant. The regional difference between the LUM in CZs declined because the EI grew from 0.680 to 0.690. An analysis of the EI for the analysed region with CLC2006 data demonstrated similar values for Czechia, Poland, and Slovakia (0.650, 0.653, and 0.657, respectively), and a lower value for Hungary (0.628). According to CLC2012 data, the EI in these countries grew, with Hungary leading the pack with 0.633. Therefore, the EI reached higher values for the LUM in FUAs than for entire countries in the investigated period.

The DI for aggregate FUAs in individual states reached from 0.149 for Hungary to 0.199 for Sweden in 2006. In 2012, the DI grew only in Slovakia by 0.002. In Poland, it fell by 0.003. In the other countries, it remained constant in 2012. The DI for aggregate FUAs assumed values below 0.3 in all countries, which means that their FUAs are not dissimilar. The highest DI values in 2006 and 2012 were identified for UCs, which ranged from 0.157 for Slovakia to 0.343 for Hungary in 2006. Here, the greatest increase in the DI was calculated for 2006 for Czechia (0.02), while the largest decrease was calculated for Poland (0.05). The UCs in Poland, Slovakia, and Czechia are not dissimilar on a national level. Only in Hungary, the DI for UCs was 0.343 in 2006 and 0.344 in 2012. Therefore, UCs in Hungary are moderately dissimilar. CZs in all the investigated countries are similar. On a regional level, the DI is the highest for UCs and exhibits no significant differences between 2006 and 2012. The DI of 0.25 indicates that UCs in the region are not dissimilar, probably due to significant urbanisation and similar LUMs.

The lowest MBI in 2006 and 2012 was identified for FUA_CZ_ and for CZs and UCs in Czechia. The highest MBI for UCs was found in Slovakia, which suggests a high similarity in their LUM to the LUM in FUA_REG_. We further identified a slight decrease in the MBI in most countries from 2006 to 2012. On the regional level, FUAs and CZs in Poland exhibited a greater LUM balance similarity to the national model than UCs in 2006 and 2012. The other countries exhibited an opposite trend: the LUMs are better balanced in relation to their national standards in UCs. The structure of LUC classes in FUA_REG_ according to UA data indicated a similar balancing as the structure of LUC classes in FUA_REG_ according to CLC data (0.970 in 2006 and 0.966 in 2012). The MBI value also indicates in this case a similar balance between the structure of the LUC in CZ_REG_ in relation to the LUM in the region.

## 4. Discussion

The investigated countries are Central European countries where arable land and agriculture intensity declined sharply in 1990–2006 [36]. CLC data show that urban areas grew further, and agricultural areas declined a little less in Czechia, Hungary, Poland, and Slovakia from 2000 to 2018. The UA data demonstrated the same for the aggregate FUA area in each country from 2006 to 2012. It is consistent with the current trends demonstrated by Bičík et al. [13], Pazúr and Bolliger [15], Wnęk et al. [8], or Taubenböck et al. [12], to name a few.

The study on the LUM in the FUAs demonstrated that the most urbanised areas, UCs, exhibited the most diversified land use. On the contrary, the most homogeneous LUM was found in the suburbs, CZs. At the same time, CZs underwent more intensive changes in the LUM than UCs. One possible scenario suggested by Antrop [3] is the urbanisation of rural areas that will have a complex and multipurpose space used intensively within a greater urban network. Therefore, the landscape in CZs may become less varied, and resulting urban areas may be more diversified at first. The diversity will fade as the areas become more developed and other LUC classes are excluded from the LUM. Dorning et al. [4] believe a population increase and urbanisation to be the primary forces behind this change in land use patterns and urbanscape. The suburban populations of the investigated countries grew while urban populations declined in the study period (Figure 1). It is also reflected by the inhibited entropy in UCs and its growth in CZs. The first areas to be built-up in CZs are those near the city boundary and a road network [37]. It is because sites that are easy to access by road are the first to be zoned as potentially residential in zoning plans. As the population moves from remote urban areas and external CZs closer to UCs, areas of FUAs near their boundaries may stop developing [38,39]. Fragmentation of arable land in CZs due to urbanisation resulting from a shift in the function towards a residential area for commuters is detrimental to its agricultural value. It will then lead to the abandonment of agriculture in CZs followed by the conversion of arable land into other land-use types. The expansion of urban fabric into adjacent rural areas turns into urban sprawl, which may then lead to a siphoning effect and outflow of workforce from rural to urban areas [40,41].

Another CZ development scenario that could account for the increased entropy in the suburbs and high MBI values is the creation of economic zones in former agricultural CZs. This phenomenon is driven by the accessibility and availability of buildable land. It makes CZs attractive areas for projects and functions that cannot be realised on developed urban land. Kopecká et al. [42] identified this phenomenon in FUA SK07 (Trnawa) in Slovakia after the Peugeot-Citroën car factory was built there on 332 ha and in SK06 (Zylina), where Kia car factories were built. The construction of a Jaguar Land Rover car factory started in 2015 in SK04 (Nitra) on 200–300 hectares of arable land is another example of this. 

Considering the above, the degree of CZ land spatial use pattern differentiation and whether these changes will be beneficial to the residents, also in terms of the quality of life and health, remains to be determined.

Regarding UCs, it is necessary to preserve the optimal share of urban green sites as well as semi-natural and natural areas there. The COVID-19 pandemic clearly demonstrated the value of access to adequate green urban areas for the well-being of urban residents [43]. Increasing soil sealing in urban areas entails issues related to poor natural water retention and local groundwater flooding. Therefore, the right proportion of green urban areas, semi-natural and natural areas, and woods in the LUM of UCs will facilitate proper rainwater management [44,45]. Local decision-makers can use local zoning plans to control soil sealing in UCs. Still, as case studies demonstrate [46], urban areas are not completely covered by local zoning plans. It is necessary to maintain a high share of vegetated areas in FUAs, especially UCs, to facilitate climate change adaptation and heat island-effect prevention through sustainable development policies [47]. The policies should provide for the increased and evenly distributed presence of vegetated areas in UCs that can buffer rainwater and prevent the adverse impact of climate change. The EI value for UCs was stable, which may be indicative of the protection of vegetated areas and more careful LUM management in cities. Furthermore, more in-depth analyses of the spatial distribution of green and blue areas in FUAs are necessary as the present research focuses on the quantitative approach. In addition, such analyzes can also be performed by considering land use changes in relation to appropriately constructed buffer zones in relation to, for example, the center of a given unit [48,49].

The analyses do not indicate that LUMs in FUAs of countries of similar socioeconomic and historical conditions, such as Czechia, Hungary, Poland, and Slovakia are much different. The indicators that quantify the LUM, the EI, DI, and MBI were similar in all the countries in all investigated variants. Therefore, the entire region exhibits no significant land-use variation in FUAs. Considering the GDP per capita as a determinant of the LUM, there were no significant differences in its values in the investigated areas, so no significant impact on LUM changes was found. However, the study did not consider detailed local LUM characteristics driven mostly by local factors and linked directly to the quality of life and health of the residents [50,51]. Moreover, LUM changes are a long-term process, so more time points need to be considered for more in-depth conclusions. Therefore, as current and verified LUC data become available, the investigation should be continued, if not also to verify the present hypotheses regarding the LUM on a national level in a selected region.

Changes in land use in urbanized areas can be caused by many factors, among which demographic, economic, policy or geographical factors are mainly indicated [52]. Among the demographic factors, the most important are population growth, population density, and migration of people from rural to urban areas [53,54,55]. The influence of these factors on LUM changes was also noted in this study. In all the analysed countries, an increase in the percentage of the population living in urban areas was observed at the expense of the population in rural areas. This situation may, in turn, be the result of agricultural transformation, accompanied by the phenomenon of abandoning agriculture and migration of people from rural to urban areas in search of jobs. At the same time, recently we have been dealing with an improvement in the health of the population and the well-being of the family and the individual. This will be accompanied by an increase in revenues and GDP, which are classified as socio-economic factors affecting land use changes [54]. In the analysed Central European countries and in the analysed period, an increase in GDP per capita was recorded, which will be accompanied by an increase in the quality of life of the inhabitants. As a result, new, often luxurious, residential districts are created, for example in CZs or on urban peripheries [56]. This is accompanied by the phenomenon of the so-called second homes, consisting in investing capital in real estate located on the outskirts of the city by residents living and working in UC [52]. Another important factor affecting LUM, in particular within the FUAs, is the transport infrastructure. The construction of a communication network will favor settlements along the roads in the CZs due to the lower land value than in the UCs. Thus, the construction of roads favors the growth of built-up areas in CZs [37]. In the considered cases of the Czechia, Slovakia, Poland, and Hungary, one more factor that has a significant impact on the expansion of urbanized areas should be noted, namely the industrial development resulting from the creation of capitalist economies in these countries after 1989. In the countries under consideration, the sources of capital are primarily foreign investment. Thus, the impact of this factor on the shift of the border of urbanized areas to suburban areas was noticed in the case of Slovakia, where in Nitra, Trnava and Zylina, industrial investments in the form of car factories were located in agricultural areas. In the case of factors influencing the LUM, attention should also be paid to the factors of the spatial policy applied in a given area and the applicable regulations [57]. In the countries of Central Europe, or those belonging to the EU, spatial policy is focused on sustainable development, and in the case of urbanized areas, spatial policy is focused on ensuring high-quality public space. In recent years, and in the regions under consideration, an increase in the importance of conducting a proper spatial policy at local levels has been noticeable. In particular, the construction of new or modernization of existing communication networks is noticeable, aimed at e.g., relieving residential areas from everyday traffic difficulties related to public transport and transport. Nevertheless, the effects of today’s spatial policy in the context of LUM will be noticeable only in the future.

Therefore, several implications for the current spatial policy can be formulated. In the case of urbanized areas, special attention should be paid to the protection of the environment and resources, including the valorization of green areas. In particular, the spatial policy conducted in the UCs should take into account the care for the appropriate spatial pattern of public green areas, while ensuring easier access to these areas for residents. Thus, within the FUAs, attention should be paid to shaping the appropriate pattern of public green areas in terms of the macroscale and the pattern of biologically active areas in the case of the microscale. On the other hand, in the case of CZs, spatial policy should focus on designating reserve areas built exclusively in the vicinity of roads. In particular, care should be taken to ensure an even formation of buildings, first in the first and then in the second line of buildings in CZs. At the same time, a policy of protecting agricultural land, forests and semi-natural areas should be pursued in the CZs. Therefore, the possibility of consolidating land in order to create clusters of agricultural areas should be considered, which may be conducive to the rational management of agricultural space and the reduction of agricultural production costs, and thus increase the profitability of cultivation in the CZs. As part of the spatial policy, protection of the best quality agricultural land as well as forest complexes and meadows against development should also be taken into account. This will allow for obtaining the highest possible yields from agricultural land and preserving the biodiversity of flora and fauna. At the same time, it is recommended to develop coherent and optimal urban planning guidelines for the creation of economic zones in CZs, taking into account the care for environmental factors.

## 5. Conclusions

To the best of our knowledge, our results are the first such work for Central Europe and FUAs with UA data that offer a better spatial resolution than CLC data. The area of class 1 grew by 0.70% in the aggregate area of the FUAs while in the aggregate area of the investigated countries, the class 1 area grew by 0.13%. The analyses suggest that the FUAs from the investigated region underwent greater entropy changes than the entire EU from 2006 to 2012. Hence, a region of countries with similar socioeconomic conditions, that is Czechia, Hungary, Poland, and Slovakia has a more heterogenic land use in FUAs than the land use in Europe. We noted slightly greater dissimilarities in UCs than CZs in the region. The identified was negligible, or, perhaps, no changes in entropy in the UCs over the study period should result from the effort to ensure sustainable development of the areas and protect land in classes 2 and 3 from conversion into class 1, leading to a heterogeneous LUM in the UCs. Using the MBI, we demonstrated that the UCs of some FUAs have well-balanced LUMs similar to a LUM developed through the pressure of a high population density typical of capital cities. Therefore, one should assume that the LUM in these FUAs will be preserved due to the intensification of built-up areas rather than their expansion as the populations grow. This suggests that some UCs have reached the optimal LUM for their respective populations. It can be accounted for by stabilised urban population growth after a boom and population movement from cities to urbanised fringes. Therefore, entropy in CZs grew only slightly, which will be due to an increase in class 1 at the expense of class 2 caused by inflation in residential developments and commercial zones.

## Figures and Tables

**Table 1:** Aggregation of LU classes for UA2006, UA2012, CLC2006, and CLC2012 data.

	UA Major LU Classes			
	1—Artificial Surfaces	2—Agricultural Areas, Semi-natural Areas and Wetlands	3—Forests	5—Water
UA2006 classes code	1.1, 1.2, 1.3, 1.4	2	3	5
UA2012 classes code	1.1, 1.2, 1.3, 1.4	2.1, 2.2, 2.3, 2.4, 2.5, 3.1, 3.2, 3.3, 4	3.1	5
CLC2006 and CLC2012	1.1, 1.2, 1.3, 1.4	2.1, 2.2, 2.3, 2.4, 3.2, 3.3, 4.1, 4.2	3.1	5.1, 5.2

**Table 2:** The entropy index, dissimilarity index, and multidimensional balance index on the national and regional level.

	Country	Poland		Slovakia		Czechia		Hungary		Regional	
Measure	Area Type	2006	2012	2006	2012	2006	2012	2006	2012	2006	2012
EI	FUAs	0.725	0.736	0.714	0.720	0.742	0.749	0.658	0.666	0.718	0.718
	UCs	0.837	0.835	0.845	0.847	0.817	0.820	0.813	0.814	0.836	0.837
	CZs	0.684	0.697	0.707	0.715	0.618	0.627	0.675	0.681	0.680	0.690
DI	FUAs	0.175	0.172	0.199	0.201	0.150	0.150	0.149	0.149	0.188	0.186
	UCs	0.207	0.202	0.157	0.157	0.207	0.209	0.343	0.344	0.254	0.255
	CZs	0.176	0.175	0.205	0.208	0.149	0.148	0.141	0.140	0.192	0.190
MBI	FUAs	0.995	0.994	0.973	0.974	0.901	0.903	0.929	0.928	0.970	0.966
	UCs	0.967	0.964	0.982	0.984	0.930	0.928	0.943	0.938	0.805	0.797
	CZs	0.994	0.994	0.973	0.974	0.889	0.890	0.922	0.922	0.982	0.986

## Data Availability

Land use and land cover datasets used in this analyses are available at: CORINE Land Cover: https://land.copernicus.eu/pan-european/corine-land-cover (accessed on 5 September 2022). Urban Atlas: https://land.copernicus.eu/local/urban-atlas (accessed on 5 September 2022). The used data of population and GDP in Europe are available at: https://ec.europa.eu/eurostat/web/main/data/database and licensed under the Creative Commons Attribution 4.0 International (CC BY 4.0) licence. (accessed on 5 September 2022).
